# Biodegradable carboxymethyl cellulose based material for sustainable packaging application

**DOI:** 10.1038/s41598-020-78912-z

**Published:** 2020-12-15

**Authors:** Jayachandra S. Yaradoddi, Nagaraj R. Banapurmath, Sharanabasava V. Ganachari, Manzoore Elahi M. Soudagar, N. M. Mubarak, Shankar Hallad, Shoba Hugar, H. Fayaz

**Affiliations:** 1 Biomaterials Laboratory, Centre for Material Science, KLE Technological University, Vidyanagar, Hubballi, 580031 India; 2 School of Mechanical Engineering, KLE Technological University, Vidyanagar, Hubballi, 580031 India; 3 Extremz Biosciences Pvt. Ltd., KLE CETEI Startup street, KLE Technological University, Vidyanaga, Hubballi, 580031 India; 4 Department of Mechanical Engineering, Faculty of Engineering, University of Malaya, 50603 Kuala Lumpur, Malaysia; 5 Department of Chemical Engineering, Faculty of Engineering and Science, Curtin University, 98009 Miri, Sarawak Malaysia; 6 Department of Agriculture Engineering, Horticulture College, Koppal, University of Horticulture Sciences, Bagalkot, 583233 India; 7 Modeling Evolutionary Algorithms Simulation and Artificial Intelligence, Faculty of Electrical and Electronics Engineering, Ton Duc Thang University, Ho Chi Minh City, Vietnam

**Keywords:** Biotechnology, Hydrology, Energy science and technology

## Abstract

The main goal of the present work was to develop a value-added product of biodegradable material for sustainable packaging. The use of agriculture waste-derived carboxymethyl cellulose (CMC) mainly is to reduce the cost involved in the development of the film, at present commercially available CMS is costly. The main focus of the research is to translate the agricultural waste-derived CMC to useful biodegradable polymer suitable for packaging material. During this process CMC was extracted from the agricultural waste mainly sugar cane bagasse and the blends were prepared using CMC (waste derived), gelatin, agar and varied concentrations of glycerol; 1.5% (sample A), 2% (sample B), and 2.5% (sample C) was added. Thus, the film derived from the sample C (gelatin + CMC + agar) with 2.0% glycerol as a plasticizer exhibited excellent properties than other samples A and B. The physiochemical properties of each developed biodegradable plastics (sample A, B, C) were characterized using Fourier Transform Infra-Red (FTIR) spectroscopy and Differential Scanning Calorimetry (DSC), Thermogravimetric analysis (TGA). The swelling test, solubility in different solvents, oil permeability coefficient, water permeability (WP), mechanical strength of the produced material was claimed to be a good material for packaging and meanwhile its biodegradability (soil burial method) indicated their environmental compatibility nature and commercial properties. The reflected work is a novel approach, and which is vital in the conversion of organic waste to value-added product development. There is also another way to utilize commercial CMC in preparation of polymeric blends for the packaging material, which can save considerable time involved in the recovery of CMC from sugarcane bagasse.

## Introduction

Literature lauds that if the plastic materials not incinerated properly yet again left littering around in the ecosystem^1^. As per the report from Olunivi et al., in 2018, about 300 million metric tonnes of conventional plastic and out of which 50% will be discharged directly into the environment without sorting^2^. Recycling of plastic is not that easy, there could be a problem with the mixed plastic pollutants, and the results from these wastes may not be suitable to obtain post-consumer products. The worldwide demand for bioplastic as an alternative for conventional plastics has augmented by considering their nontoxicity, biocompatibility, renewability, and biodegradability properties^3^. Biobased plastics can be degraded by means of microbial processes, and they are commonly produced using different raw materials (proteins and polysaccharides), which are mostly derived from plants (cellulose-based plastics and starch derived plastics), and microbial source [polyhydroxyalkanoates (PHAs) and polylactic acid (PLA)].

The size of the plastic material is very critical due to its potential hazardous character to each individual, communities, and whole ecosystem^4,5^. Therefore, an uncontrolled increase in population with excessive non-renewable resource exploitation resulted in cumulative waste volume. At present, there are few methods involved in partial elimination of these wastes, for example, landfills and ocean overflow of numerous materials, some among them can degrade in a stipulated time, while other debris cannot be degraded for several hundreds of years.

Alternatively, the bio bags assumed to be not much distinctive from conventional plastic bags^6^. They have provided with all these associated issues to conventional plastic, as an alternative biodegradable plastic holds excellent characteristic properties and is more reliable nowadays. The objective of the present work is to develop cellulose-based bioplastic material. It could be one of the best possible solutions to the gap created due to the ban on conventional plastic around the globe. Biodegradable plastics conspicuously derived from renewable biomass resources such as vegetable waste, fruit waste, biopolymers, and microorganisms^7^. Biodegradable plastic prepared using agricultural by-products, as well as from used plastic waste with the help of microbial degradation methods. Biodegradable plastics can be broken down into a smaller part through biological treatment either by applying aerobic or anaerobic techniques, and the process is generally defined based on the types of bioplastics such as starch, cellulose, biopolymers-based materials.

Literature reveals, most of the researchers used starch-based films for food packaging applications due to the easy manipulation and their ideal properties^8–12^. Many other polysaccharides such as alginate, pectin, chitosan have significant gas barrier properties, and the films developed by using these polymers ideally flexible, transparent, stable, and are also resistant to fats and oils^13–15^. In recent years global demand for biodegradable plastics based on sugar, starch, and cellulose has increased by up to 600. Apart from the above reports, in past years, many insights on the development of polymeric nanocomposites for packaging^16–18^, biomedical^19–24^, aeronautics^25^ and automotive^26^ applications. Polymeric nanocomposites due to their lighter molecular weights than synthetic plastic material and also has higher mechanical, rheological, thermal, and biodegradable properties^27,28^. However, having excellent market opportunities and proven advantages, the accessibility of nanocomposite materials is still a long way to enter into the present market^29^. Subsequent with these reports, researchers^30–32^ were also discovered the applications of antimicrobials, antioxidants, and wound healing activities of these polymer mixtures. Development of biodegradable starch-based foams incorporated with grape stalks for food packaging^33^.

The current requirement of food packaging materials in different sectors is enormous. Food must be wrapped with the packaging material to keep food safe by avoiding possible contamination from the environment. It is a vital process to preserve food until delivery to the buyer. The significant factors influenced by the package materials depend on the mechanical strength, permeability to oil, water, oxygen, microbial action, etc. during storage and their distribution. Likewise, other essential factors are concerning recyclability, material costs, disposable nature, and sustainability^15^.

In upcoming years these biodegradable polymers are much anticipated to replace conventional polymeric goods in packaging applications^33–35^. Though it is crucial to characterize the biopolymers permeability to aroma and flavors compounds, limited work has carried out^36,37^. Though there is a massive demand for cost-effective, consumer, and environmentally friendly sustainable plastic alternatives, however, there is scarce in its supply. In this context, the present work could fulfill the immediate requirement. Since because the considered work performed mainly uses renewable resources, mostly sugar cane bagasse could be an innovative approach to convert waste into wealth technology.

## Results and discussions

### Development of CMC based material

During the process, CMC s added to the mixtures of gelatin contained solution that creates active site within the polypeptide chains and thus, forms hydrogen bonding and electrostatic interaction amongst the polymers. The increased concentration of CMC tends to provide potential active sites for cationic-anionic interaction between these polymers. Therefore, the intramolecular and intermolecular interactions have led to stable film formation, and accordingly, exhibited better flexibility.

### Biodegradation of the developed material

As per the report from Bella et al.^38^, the degradation phase of CMC correlated with degradation of the carbonyl groups and extremely interactive side chains. Because of the crystalline nature of CMC, the association between gelatin solution containing agar can improve the thermal stability of the polymeric film. Hence, the thermal breakdown of CMC-gelatin blend-based film has turned to be higher temperature stability than the solitary gelatin films.

Nowadays, one can understand that the critical factors for ideal packaging materials are their mechanical strength concerning to holding the materials intact and equally the biodegradability, means that it has to disintegrate or decompose after the use. Meanwhile, it should not cause any contamination or pollution either in soil, water and air. Interestingly, the present research effort towards the development of ideal packaging material showed good tensile strength as well as an environmentally friendly nature.

Few impressive contrasting results obtained concerning to the effect of glycerol on film formation. In a previous study, Fatma et al.^39^ have reported that beyond 15% of the glycerol presence was used to induce flexibility of the composite material and concluded that an increase in glycerol concentration directly contributed to the plasticizing effect. However, in the present work, limited quantity i.e. about 2% glycerol, has positively impacted the flexibility of the developed material. From the above comparison, it can be inferred that the blend formed using starch and polylactic acid and starch must require higher concentrations of glycerol and have deliberated a positive impact on the mechanical properties of the film. Nevertheless, in the present study, both agar and a small amount of glycerol played an essential role in the enhanced flexibility of the film.

The results presented below confirming its characteristic features compatible concerning the biological degradability. The percentage of biodegradation employing microbial activities have shown in Table 3. During the experiment, the initial quantity of samples A, B, and C took for the experiment and the weights after 3, 5, and 7 days of degradation, respectively. After stipulated time intervals, bioplastic samples in the soiling conditions observed, surprisingly, there was a significant reduction in their thickness and weight. The results of three different as follows, within the first three days of incubation, there was 13.02%, 27.62%, and 29.32% reduction in samples A, B, and C, respectively. Similarly, after 5 and 7 days 88.21%, 81.95%, 90.23% and 95.08%, 91.66%, 96.41% of reduction in the samples were occurred. From this experiment, concluded that all the samples prepared were biodegradable within 7–10 days. The biodegradation is much faster than the biodegradable plastics developed using PVA-gelatin films, as reported by Sajjan et al.^40^ (Table 1).

**Table 1:** Shows the solubility test results of all samples soaked in a different solvent medium.

Sl. no	Solvent	Samples	Insoluble	Partially soluble	Completely soluble
1	Ammonia	A	–	–	**√**
		B	–	**√**	–
		C	–	–	**√**
2	Acetic acid	A	–	**√**	–
		B	–	**√**	–
		C	–	–	**√**
3	Chloroform	A	–	–	**√**
		B	–	**√**	–
		C	–	–	**√**
4	Acetone	A	–	–	**√**
		B	–	**√**	–
		C	–	–	**√**
5	Methanol	A	–	**√**	–
		B	–	**√**	–
		C	–	**√**	–
6	Sulphuric acid	A	–	–	**√**
		B	–	**√**	–
		C	–	**√**	–
7	Orthophosphoric acid	A	–	**√**	–
		B	–	**√**	–
		C	–	**√**	–
8	Ethanol	A	–	**√**	–
		B	–	**√**	–
		C	–	**√**	–
9	Water	A	–	**√**	–
		B	–	**√**	–
		C	–	**√**	–

### Sample swelling and solvent solubility studies

In determination of the solubility of the developed films, these films samples, in real time initial quantities of the samples A, B, and C taken for the swelling test and the final weight after swelling the samples through different media for 2 h (Table 4). From the sample solubility table indicated that there is an increase in the weight of the samples when treated with the water and chloroform (Table 1). However, when the samples treated with the methanol, it was surprise, instead of losing weight and bioplastic became harder. All the samples prepared were either wholly or partially soluble in the above solvents used, which are desired results for the biodegradable polymer blend preparation.

### Environmental compatibility and characteristics of packaging

Many of the Indian states have imposed the ban on single-use plastics, and in recent times USA and Rwanda have banned the use of plastic bags, microplastics, and styrofoam. As a case study, the California microbead ban was accepted in 2015. As an outcome, the ban delivered the maximum protection against the plastic microbead pollution in the country. Accordingly, the bill offers companies to come up with natural alternatives like apricot pits and walnuts husks^2^.

Though, it is difficult to clean up the plastic (specifically micro-sized) from the environment due to their small size and less distinctiveness. However, recent advancement in biotechnology has delivered reliable and much favorable approach to overcome the challenges of plastic pollution that occurred around the world^2^.

Disposal of waste is also a significant factor in various farms. There are several reports in the past few years, the process of burning the agriculture-based waste has led to the seasonal air pollution exclusively in the southeast of Asia, mainly Singapore, Indonesia, and Malaysia. The extended air pollution results in public illness also affect the socio-economic status of the people. Identification of naturally available constituents is another area of research in which could strengthen the cost-effective biomaterials development and also helpful addressing the field of waste management. With emphasis on “waste to wealth,” the present work reflected the conversion of bagasse waste to the useful byproducts such as cellulose pulp and was further processed to yield carboxymethyl cellulose. To isolate the carboxymethyl cellulose technique described by Chia et al.^41^ was used. For about 50 g of sugar cane bagasse, an extract of 6.18 ± 0.1 g of carboxymethyl cellulose obtained, and further, these extracted celluloses utilized in the development of bioplastic blends.

The concentration of CMC content of 1.2 g kept constant (with fixed gelatin (1.15 g), and agar contents of (0.55 g) 1.0%, respectively) and variability of the percentage content of glycerol [sample A (2.5 ml), sample B (1.5 ml) and Sample C (2.0 ml)] for 100 ml sample solution. The thickness of the composite blend film did not change significantly, however, the tensile strength of the film was increased with an increase in the concentration of glycerol sample A 13.2 ± 0.28 MPa to sample C highest 19.81 ± 0.41 MPa and then after decreased to 14.68 ± 0.28 MPa of sample B (Table 5). The results are related with the previous report Inyoung et al.^42^, reported that the tensile strengths of PE (polyethylene) and PP (polypropylene) were 14.76 MPa and 26.96 MPa, respectively. However, in our study, we have observed maximum tensile strength corresponding to sample C that is 19.81 ± 0.41 MPa, further film tensile properties can be enhanced in future studies.

More particularly, according to the present report, the tensile strength was first decreased, later increased, and lastly, decreased again, with the maximum value reaching 9.56% ± 2.17%. Due to the lower CMC content, which was 15% of the overall mass of the composite film sample, the sample thickness did not alter much with the increased addition of CMS. Yet, in CMC, there is an internal sugar ring structure, which has an excellent skeleton effect as a composite structure, and that can improvise the internal structural stability of the composite blend film; therefore that will help in increasing the ductility and tensile strength of the composite sample^43^. However, in the present study, instead of varied concentration of CMC, the percentage of glycerol content (1.5%, 2% and 2.5%) were selected for the current experimental.

Although the ductility was good at 1.5% glycerol, the tensile strength diminished. The water uptake and moisture permeability of the composite is associated with the hydrophilic properties of the molecules. Ebrahimi et al.^44^ have proved that the WVP is one of the leading indicators in circumventing the mass transfer among the food and the surrounding environment preview of the membrane. Moreover, Kanmani et al.^45^ recommended that WVP is much affected by various factors such as hydrophobic properties, film thickness, crystallinity and other integral components of the film. The precursors used in preparing the composite film consists of numerous hydrophilic groups, that exclusively increases the permeability coefficients of all combinations of substrate, thereby restricting their food packaging applications to lower water-containing foods. The CMC, gelatin, and agar are well soluble in water. The addition of little quantity of the CMC has slight effect on its moisture permeability coefficient and water absorption.

**Figure 1:**
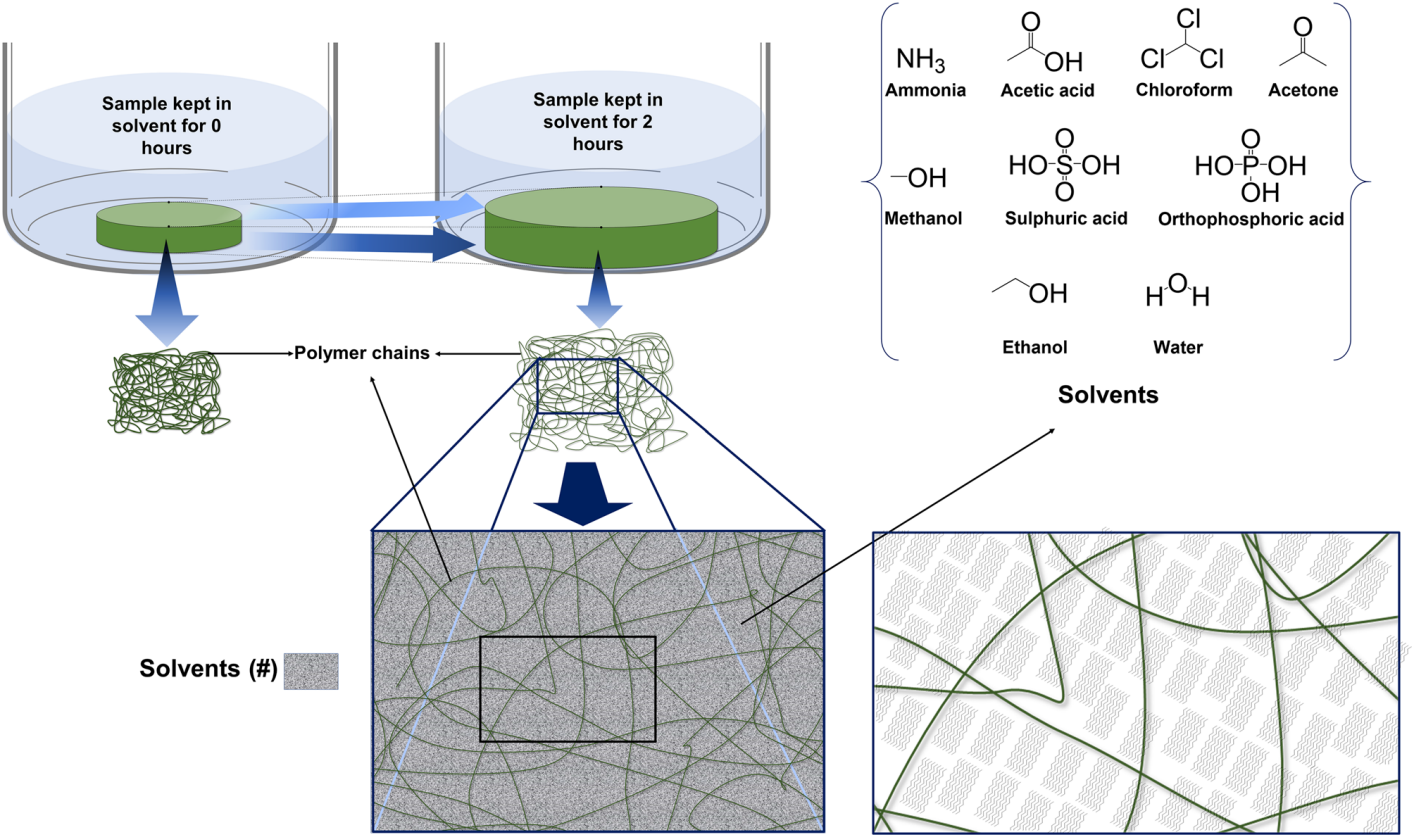
Mechanism of permeability and solubility properties of composite film.

The CMC structurally consists of cellulose fibers that have given a tortuous path for the water vapor to penetrate the composite films^46,47^. The addition of CMC into the mixture of solution (consists of gelatin and agar) which creates an active site in the polypeptide chains for electrostatic interaction and hydrogen bonding among the polymers. An increase in the amount of CMC dictates the increased potential active sites for cation and anion interactions amongst polymers. This specific interaction has outcome as a more compressed film formed between the polymeric composites, which bears a stronger film along with improved flexibility. Yet, because of the presence added quantity of CMC, the hydrophilicity of the composite film could exist as shown in Fig. 1. As per the previous report, the succeeding application of the CMC based composite films can be used to preserve fresh fruits due to higher ability of moisture absorption and permeability that helps to absorb the moisture released through the respiration process by the fresh fruit and can resist food spoilage. The bioderived composite film can be utilized in food preservation and which improves the safety as well as the quality of foods. Bajpai et al.^48^ reveal that the moisture absorption rate of the film is associated with the content of CMC, whereas the film has strong moisture permeability. This property is mainly because CMC has a surface hydroxyl group and which can be easily absorbed by water molecules and reduces the CMC composite film to become hygroscopic. Once the water vapor enters into the film matrix, the fluorene group of the cellulose chain does not interact by a invading water molecule due to the strong internal triple bond interaction, and that display good moisture permeability. This property is very much important in preservation and also vital in extending the shelf life of fresh fruits.

Another important property, such as oil permeability of the film, is concerned with the quantity of the lipophilic groups existing in the composite film. Ebrahimzadeh et al.^49^ and Wenting et al.^50^ have reported the CMC based composite film to possess lipid and oxygen barrier properties. Yet, related to the small concentrations of CMC in the composite film, the impact of CMC on the oil resistance properties of the film is not that significant. Thus, the developed film has a low oil-repellent property that can impart appropriate packaging food with lower oil content. It is also dictating that the decrease in the light transmittance rate of the developed film.

### Surface morphology study

**Figure 2:**
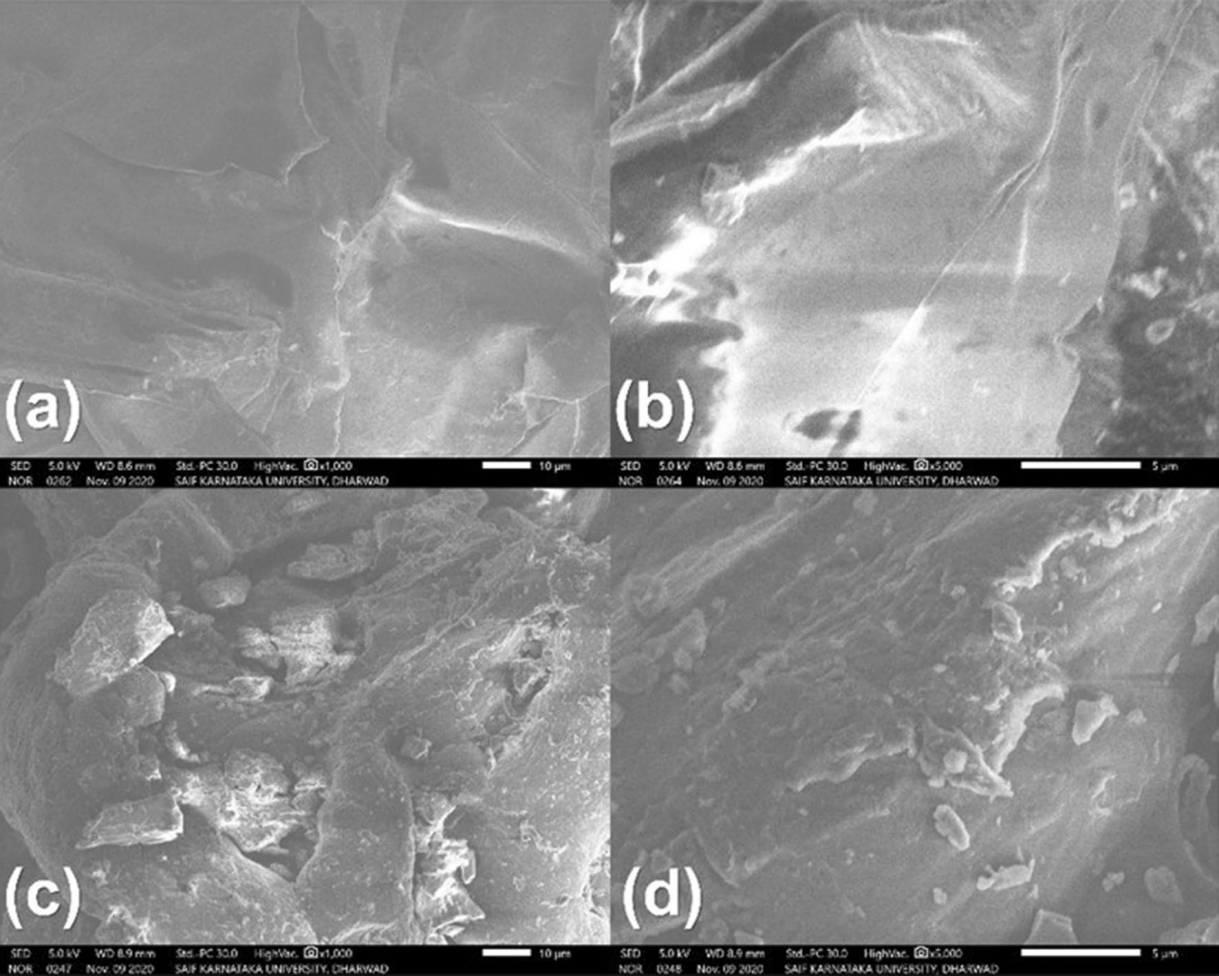
Depicting (**a**,**b**) are the Scanning electron microscopic (SEM) images of sugar can bagasse before the treatment and (**c**,**d**) represented the SEM image after the treatment of bagasse with acetic acid, sodium chlorite and other solvents.

The surface morphologies of the sugar cane bagasse before and after pretreatment were observed under SEM (scanning electronic microscope). By observing at Fig. 2 it can be concluded that the before treatment with acetic acid and sodium chlorite solutions the SEM Fig. 2a,b indicated surface was much more intact, however after the pretreatment with different solvents bagasse loses its ingrity and forms into separate polymers shown in Fig. 2c,d.

### FTIR, DSC, and TGA analysis of the CMC based material

**Figure 3:**
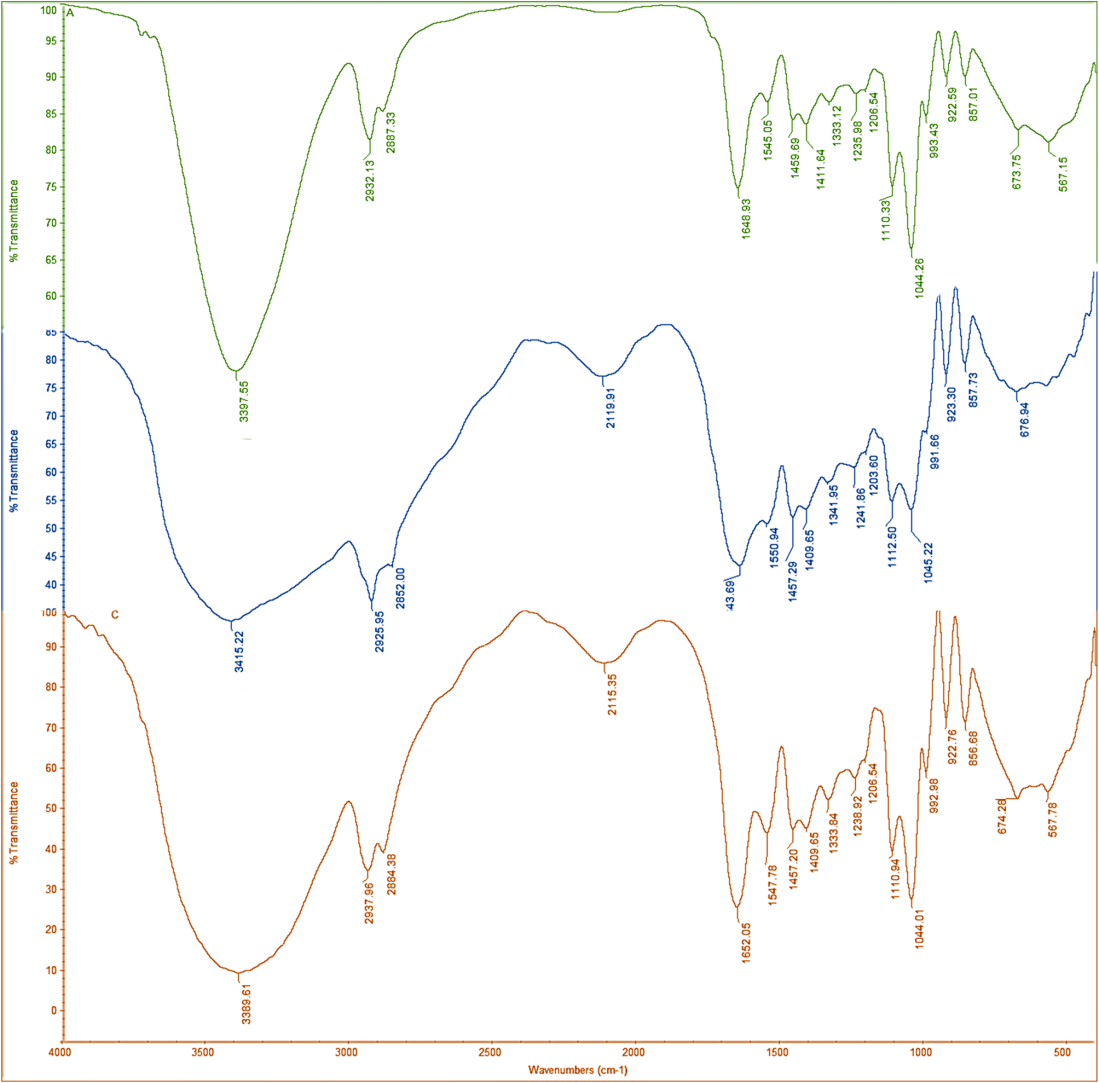
Fourier Transform Infrared Spectroscopy (FTIR) results of sample A-Green color, sample- B-Blue color, and sample C-Maroon color.

Fourier transform infrared spectroscopic (FTIR) analysis of, sample A, B, and C shown in Fig. 3 carried out to confirm the functional properties which correlate functional group and structure of composite blended bioplastics. FTIR identified effects of molecular interactions involved between gelatin, CMC, and agar on a film’s structure. Figure 3 indicated the FTIR spectra result for all formulations, which evidenced a few important functional group patterns observed.

As per the previous report of Jahit et al.^51^ (i) Ranging between 3200–3400 cm^-1^ amide A and free water; (ii) The amide I group, which signifies C=O or COO coupled with hydrogen bonding (1700–1600 cm^-1^); (iii) the amide II group, N–H groups represented as bending vibrations and C–N groups (1500–1600 cm^-1^) described as stretching vibrations of; and (iv) the Amide III group relation to C–N and N–H groups of bound amide (1000–1250 cm^-1^) with a plane fluctuations. The amide I group is the most susceptible region of the protein’s secondary structure^51^. In recent years there is enormous attention on crystallization biodegradable polymer-based films. Reports of Shuya et al.^52^ have deliberated the control mechanism for the crystallization of thin biodegradable polymers films (Table 2).

**Table 2:** Effect of temperature on degradation (weight loss) of the samples.

Sample A		Sample B		Sample C	
Temperature in °C	% weight loss	Temperature in °C	% weight loss	Temperature in °C	% weight loss
43.17	4.5	47.96	3.31	38.57	4.39
79.52	17.45	96.08	11.1	74.56	14.28
192.92	31.64	224.40	27.93	207.31	33.08
307.75	69.1	261.77	37.97	249.90	44.67
372.69	78.11	294.98	50.95	273.99	52.55
441.36	83.15	310.90	55.37	306.63	64.51
487.18	84.96	415.92	69.08	397.14	75.42
517.71	85.64	487.18	73.42	468.62	79.6
692.29	87.49	692.68	76.89	649.99	82.48

**Figure 4:**
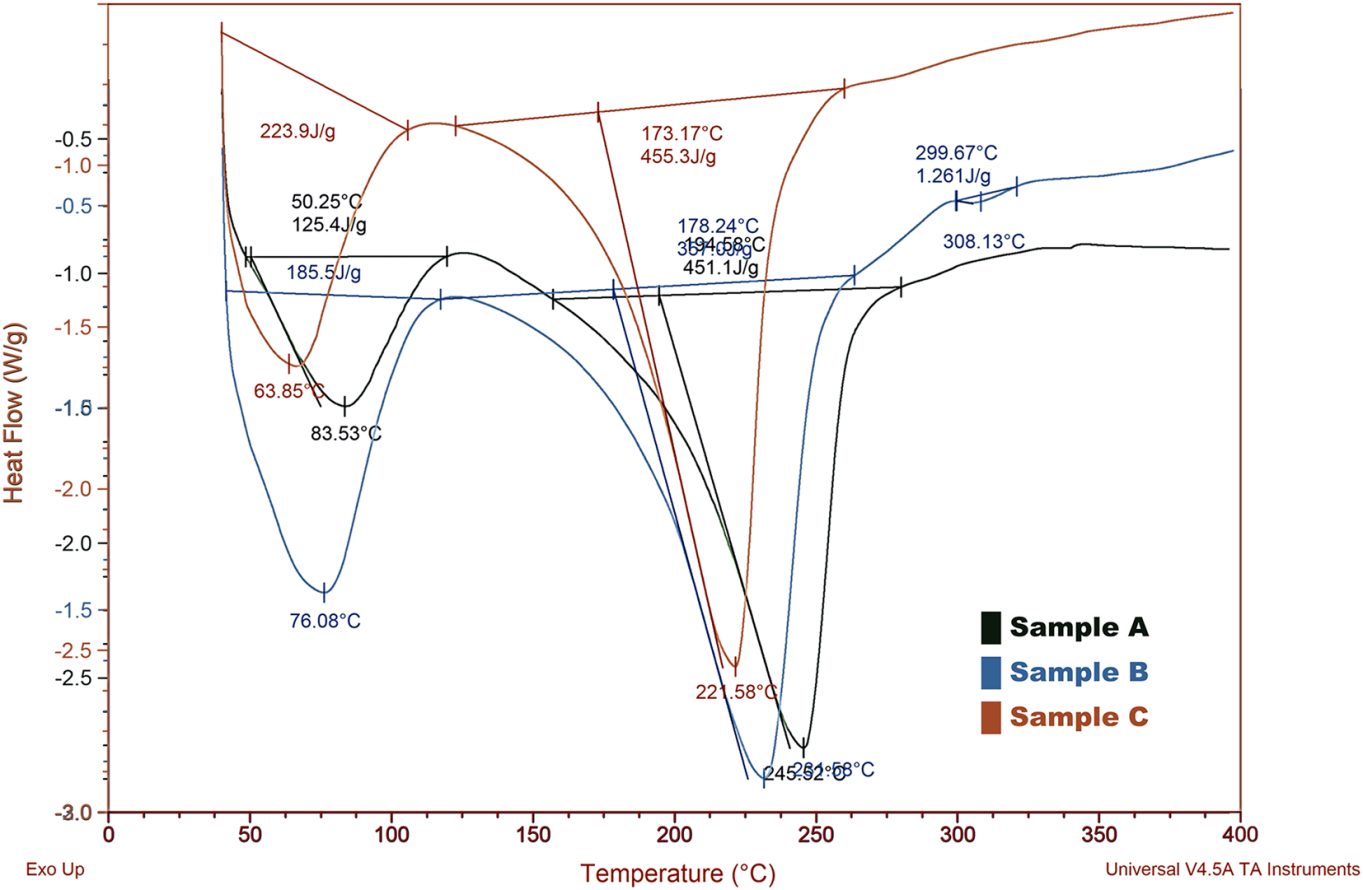
Differential Scanning Calorimetry (DSC) results of samples A, B and C.

Similarly, films in the molten state cooled until glass transition temperature (Tg). During this period, the polymeric samples lose their elastic properties and turn into a brittle one; this is because of the simultaneous change in chain mobility occurs. Heat flow versus temperature plot at different glass transition temperature indicated in Fig. 4 in which sample A, B, and C reach its glass transition temperature (Tg) at 50.25°, 76.08°, and 50.25 °C, respectively. At higher glass transition temperatures, the polymeric chains have shown high mobility, and have received sufficient energy for stepwise arrangements and to form into a crystal. As crystallization is the exothermic process, hence heat is being released to the environment. Relatively less heat was provided to the sample pan and reference pan and the samples A, B, and C reach their crystallization temperature (Tc) at 63.65˚C by releasing heat fusion of 223.9 J/g (Tables 3, 4, 5).

**Table 3:** Indicated the biodegradability of all three samples.

Sample name	Initial weight (g) (zero days)	Grams weight loss after 3 days of incubation and % of degradation	Grams weight loss after 5 days of incubation and % of degradation	Grams weight loss after 7 days of incubation and % of degradation
A	0.4114	0.0536 g (13.02%)	0.3093 (88.21%)	0.0283 (95.08%)
B	0.1524	0.0421 (27.62%)	0.0828 (81.95%)	0.0148 (91.66%)
C	0.3482	0.1021 (29.32%)	0.2121 (90.23%)	0.0215 (96.41%)

**Table 4:** Indicating the swelling test results in different solvent system.

Samples	Medium	Quantity of medium taken in ml	Initial weight of the samples in g	Final weight of samples obtained in g
A	Water	20	0.0197	0.1525
	Chloroform	5	0.0199	0.0654
	Methanol	5	0.0178	0.0073
B	Water	20	0.0152	0.2582
	Chloroform	5	0.0127	0.0310
	Methanol	5	0.0135	0.0102
C	Water	20	0.0173	0.1511
	Chloroform	5	0.0190	0.0338
	Methanol	5	0.0166	0.0081

**Table 5:** Few important characteristic features possessed by the developed film for the ideal packaging material.

Film sample	Tensile strength	Break strength	Water permeability	Oil permeability
Sample A	13.27 ± 0.28	8.51 ± 0.22	1321 ± 51	17.53 ± 1.02
Sample B	14.68 ± 0.29	9.16 ± 0.23	1360 ± 140	43.21 ± 2.14
Sample C	19.81 ± 0.41	0.79 ± 0.14	1143 ± 25	21.71 ± 4.16

The polymer chains can able to move freely at the melting temperature (Tm). Accordingly, they do not have any ordered arrangements as the process of melting is an endothermic process that requires absorption of excess heat. While heating also the temperature was kept constant. However, the energy added during this period could be used to crystalline regions and usually do not increases their kinetic energy gained by the chains there in the melted samples. The plotted graph of heat against temperature seems like a jump discontinuity at the melting point. in which sample C reaches its melting temperature (Tm) at 221.56 °C by absorbing heat fusion of 455.3 J/g.

**Figure 5:**
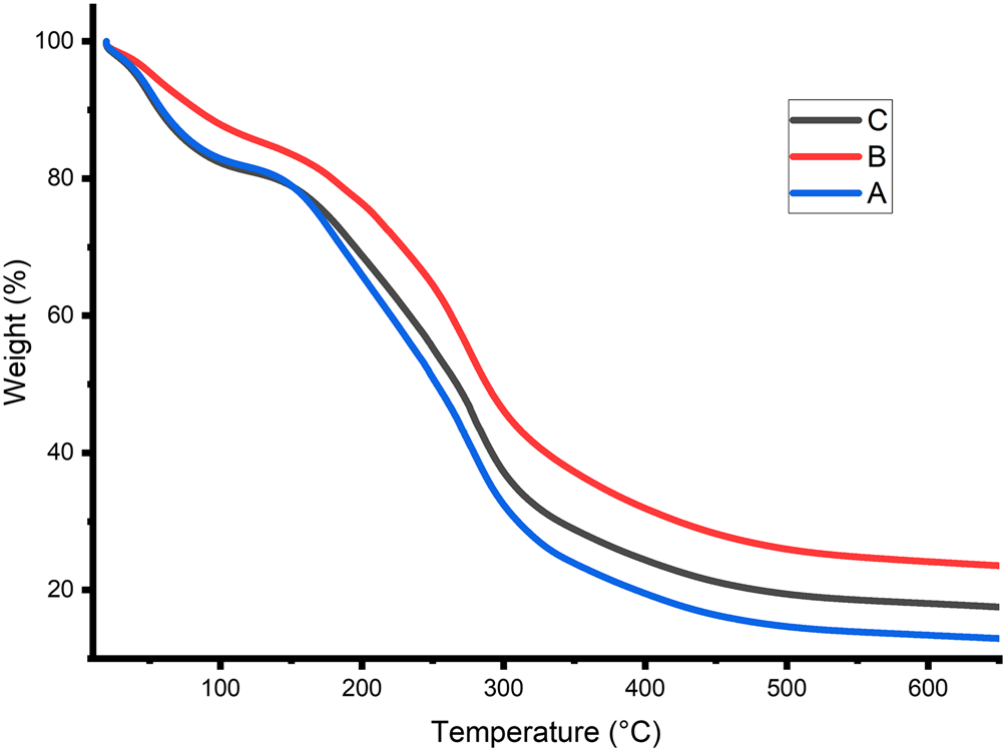
Thermogravimetric analysis (TGA) of sample A, sample B, and sample C.

Thermogravimetry (TGA) analysis conducted to measure the change in weight of a sample concerning increased, cooled, or held at a constant temperature. The significant outcomes of the TGA are polymer blends deformation concerning to the temperature, and the functional groups present in the material can be determined and enumerated. TG Analysis curves for the gelatin/ CMC blends are shown in Fig. 5 temperature for degradation of gelatin/ CMC blends are shown in Table 2. The thermogravimetric curve of gelatin/CMC clearly shows the major part of the degradation of sample A, B, C occurs in 3 stages, ranging from 43–192 °C, 192–373 °C, and 373–692 °C for sample A, 48–224 °C, 224–295 °C , and 295–692 °C for sample B, 39–207 °C, 207–274 °C, and 274–650 °C for sample C. This result could be due to the fact that the two constituents of the blend films were diffused uniformly and have been strongly combined. In addition, the residuals (at 700° C) of the blend films were increased with increasing of the CMC components, which implied that the addition of CMC contributed to thermal stability property. The thermodynamic properties of the blend films were affected directly by the partially crystalline polymer CMC^53^.

Reduction in weight of the sample shown in Table 2, Fig. 5, during first stage TG analysis could be due to loss of free water in the second stage due to evaporation of water and third stage degradation or deformation of CMC and gelatin constituents. During the third stage of the process, significant gases such as Carbon monoxide (CO) and Carbon dioxide (CO_2_), water, and some volatile compounds release with carbonaceous residues. CMC exhibited steady weight loss from 192 °C, 224 °C, and 207 °C, respectively for samples A, B, and C; this could achieved due to the conduction process leads to evaporation of water and glycerol constituents.

From the previous studies, it can be concluded that the significant disadvantages of chitosan-based films easily dissolve in acidic solution and also low flexibility^54,55^. However, polyvinyl alcohol has excellent properties such as higher hydrophilicity, chemical stability and unique film-forming potentiality required for the typical packaging applications^56,57^. Yet it needs a specific type of microorganism or properly acclimatized microbes to degrade it^58^. Concerning to above issues, the present work has accomplished a significant breakthrough that the developed biodegradable plastic is not only hydrophilic. It is also flexible, transparent, and robust; it has higher biodegradation rates when compared to existing biodegradable plastics.

## Future prospective

CMC is the most crucial derivative of cellulose and which has been used as an excellent stabilizer in the food industry and can be the best raw material for packaging material. The main characteristic feature assimilated by the CMC was concerning to its strong barrier properties against carbon dioxide, oxygen, and lipids. Therefore, it can be considered as the vital additive in making the more durable film. As described by many researchers^59–61^, CMC does have exceptional barrier properties and mechanical properties, and which display for better compatibility.

The complex interactions and heterogeneous resultants of gelatin, CMC, and agar-based on the properties of gelatin-based films demonstrated that the agar association significantly influences the physical properties of bioplastic formation. Cristina et al.^62^ have described the biodegradable/compostable package could preserve the nutritional quality of fruits compared to standard package, and also offer a potential alternative to reduce the risk of processing the nutrients. Additionally, biobased package material could cut down the environmental effluence released through plastics waste disposal, especially in the packaging segment. As the CMC is expensive by its price, there must be a economical extraction methods for the recovery of CMC is another gap where it requires huge attention from the researchers. That would yield better availability for developing relatively cheaper polymer-based products. The present work has the potential to attract more researchers in contributing to the field of agriculture waste management certainly, wealth development using waste and has a significance of breakthrough in the packaging sector.

## Conclusions

In the present work, throughout the studies, we have consistently extracted the desired CMC, a useful macromolecule present in the agriculture waste with an emphasis on the sugar cane bagasse. All the samples prepared characterized and the complex interaction between gelatin, CMC, and agar unveiled by FTIR and DSC, TGA, solubility, swelling, and biodegradation studies. These physicochemical properties of the gelatin/CMC/Agar blends of bioplastic produced to conducted attributed environmental compatibility**.** The sample C prepared using gelatin/CMC/agar with 2.0% glycerol was found best formulation and as well as optimal for potential use in food packaging applications, as it had excellent characteristic features like lowest water vapor permeability and the highest biodegradability rate as compared to other samples. Furthermore, trials are in progress to use the developed material in food packaging applications.

The current study delivers a strong basis for researchers who are working on effective waste conversion technologies. The present research involves the production of biodegradable plastic production using mixtures of CMC, which are derived from the agriculture waste along with gelatin, agar, and glycerol. By following similar processes, most of the agricultural waste, fruit waste, other organic debris can be new avenues for the researchers and scientists who are looking for marvelous materials in terms of cost, feasibility, and environmental compatibility. The current process also provides invaluable insights for the effective use of renewable sources typically seen around the environment.

## Materials and methods

### Sugarcane bagasse to cellulose pulp and carboxymethyl cellulose extraction

The cheap agriculture waste sugarcane bagasse collected from the local market of Hubballi. Further, 100 gm of the sugar cane bagasse (considered as moist weight) used for further processing. Two steps process conducted to extract the carboxymethyl cellulose from the agro-waste. As a first step, the extraction of cellulosic pulp carried out according to the method^39^. The agro-waste material was oven-dried at 80 °C ± 2 for 16 h, after the incubation, sample blended using pistil and mortar. The percentage of moisture content in the sample calculated by the difference of wet weight over the dry weight of the sample. Further, it was processed to obtain cellulose pulp, as described by Caroline et al.^63^ .

#### Cellulose pulp

Thus, obtained sugar cane bagasse (SCB) was oven-dried at 80 °C for 3 h and filtered with a laboratory mesh of 0.5 mm diameter. The SCB weighed to 100 g was treated with about 200 ml of 0.5% acetic acid, and 1% sodium chlorite in a stopper contained Erlenmeyer flask to confirm the entirely removed lignin. The mixture was then heated under a shaking water bath at 80 °C for 2 h. The cellulosic pulp was later filtered, washed with distilled water, and kept in a hot air oven at 80 °C. The crude cellulose can be harvested and further processed.

#### Carboxymethyl cellulose

**Figure 6:**
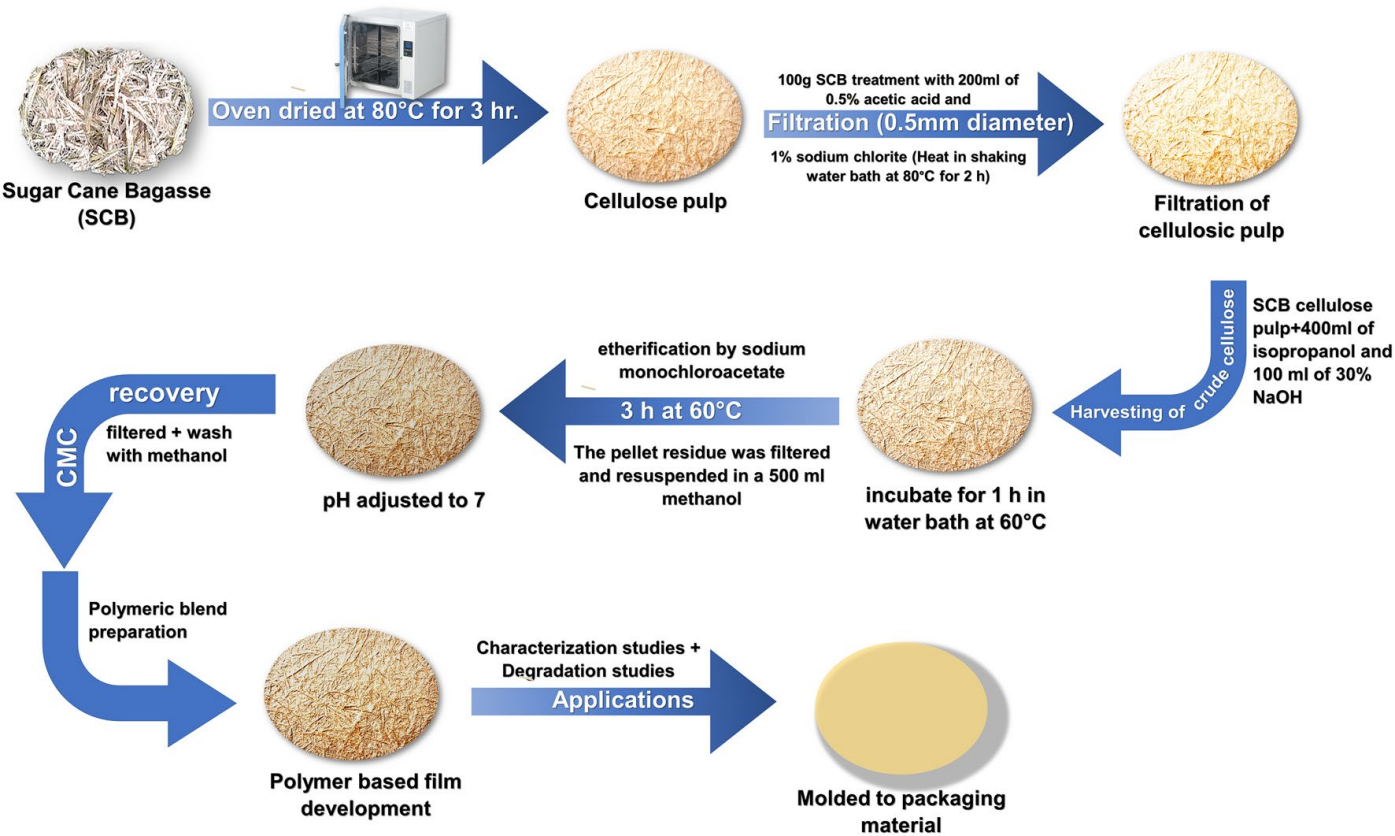
Schematic representation of the development of Biodegradable plastic for sustainable packaging.

The method followed in the recovery of CMC was carried out as per the reports^64,65^. The SCB cellulose pulp was added in the 400 ml of isopropanol and 30% NaOH of 100 ml. The mixture of the sample was incubated for about 1 h under a shaker water bath at 60 °C in order to alkalize the cellulose. After, the process of etherification was initiated along with the addition of sodium monochloroacetate, and the reaction was maintained for about 3 h at 60 °C. The pellet residue was filtered and resuspended in 500 ml methanol. The pH was adjusted to 7, using 10% of acetic acid, and left overnight. The main aim of this step was to neutralize any sort of NaOH within the mixture^66^. In the next day, the mixture was again filtered and washed with methanol, and then the remaining alcohol was removed the carboxymethyl cellulose recovered was expressed as grams per 20 g of pulp. The CMC was purified and extracted by the modified protocol as shown in Fig. 6. The CMC obtained from commercial suppliers can also be used for ease development of CMC-gelatin-agar blend package material.

### Preparation of polymeric blends

Various compositions of gelatin, carboxymethyl cellulose (CMC), and agar prepared in an Erlenmeyer’s flask contain distilled water, gelatin-1.15 g, carboxymethylcellulose-1.2 g, and agar-0.55 g with equal proportions of sample A, sample B, sample C separately with a different concentration of glycerol of sample A-2.5%, sample B-1.5%, and sample C-2.0% of glycerol, final volume of each solution maintained to about 100 mL. All these three samples were stirred on a magnetic stirrer with a temperature of 50˚C, continuously for 2 h. To attain a uniform solution. The obtained viscous solution was then cast on a petri dish /glass plate using a glass rod. The samples were oven-dried for about 24–36 h to remove the excess content of moisture, and after the incubation, carefully removed the polymeric film using a sterile blade to avoid microbial contamination.

### Environmental compatibility studies

#### Biodegradation test

Three 500 ml volume beakers filled with a garden soil sample. Samples A, B, and C were prepared using a sterile blade; then the samples were cut into small pieces and weighed, placed in 3 different beakers at a depth of 5 cm. 20 ml of water was sprinkled on the soil to enhance the soil microbial activities. Approximately 4 cm × 2.5 cm size samples taken for biodegradation experiment and weight of the samples after 3, 5, and 7 days, results recorded, and the percentage of weight reduction calculated of each sample.

#### Swelling test

Centrifugation tubes used to carry out the swelling studies. A piece of all samples A, B, and C weighed. The swelling analysis was carried out using three different solvent systems containing water, chloroform, and methanol separately. The pre-weighed film samples were kept in a solvent medium for about 2 h. The results were recorded accordingly, the gain in the weights of tested samples calculated against the pre-weighed samples.

#### Solubility test

All the samples A, B, and C were cut into small pieces and inserted into a test tube containing different solvents. Samples solubility in these solvents checked. Whether the sample is soluble, partially soluble, or insoluble were determined. The solution used is ammonia, acetic acid, chloroform, acetone, methanol, sulphuric acid, orthophosphoric acid, ethanol, and water as shown in Table 1.

#### Determination of oil permeability coefficient and water permeability (WP)

During the test, the test tube was added with 5 mL of edible oil, and then this tube was sealed with the film sample. Further, the sealed test tube was placed inverted position on the filter paper (with known weight), and the test tube kept at the center in the sidewall and left for 1 day. Subsequently, after the incubation, the filter paperweight was measured using an electronic weighing machine, and the oil permeability coefficient was calculated by following the equation:

$$P_{0} = \left( {\Delta W \times FT} \right)/\left( {S \times T} \right)$$

where *P*_0_ is the oil permeability coefficient (g·mm/(m^2^·day), ∆*W* difference in the filter paperweight (g); *FT* is the thickness of the film (mm), *S* is the area of the film (m^2^), *T* is incubation time (in days). Similarly, water permeability test was conducted along with the CMS/CS blend film and results were calculated according to the same formula; where by considering water permeability in place of oil permeability coefficient.

### Characterizations of developed bioplastic

#### Microtensile strength

A micro tensile test was conducted by following ASTM 882- 2: tensile properties of thin plastic sheeting. The tensile tests indicate the force required to break the CMC based film and also the extent of sample stretch and elongation up to breaking point. Which helps in the determination of mechanical strength bear by the sample.

#### SEM analysis

The SEM (Scanning electron microscope), JEOL, JSM-400^o^A, Tokyo, Japan, was used to determine the surface morphology and cross-sectional areas of the CMC based composite films. During the process, all the samples were blot dried completely and coated with a conductive layer (400 ^o^A) of a aluminum sheet.

#### Fourier transforms infrared spectroscopy (FTIR) test

Characterization of the developed films was done through the Fourier Transform Infrared Spectroscopy (FTIR) and obtained an infrared spectrum of absorption or emission spectra of the films were analyzed. FTIR spectrometer determines the hydrophobic properties of the membrane and also signifies the polarity of the sample at a given site.

#### DSC test

DSC analyses carried to determine the glass transition temperature of the polymeric films. All three samples were examined by using test and reference samples at the same temperature during the experiment. Where the temperature program designed according to DSC analysis, and the sample holder temperature increased linearly concerning time. However, the reference sample has a well-defined heat capacity along with a various range of temperatures to be scanned.

#### TGA analysis

Thermogravimetric analysis (TGA) of the polymer blends carried out using SDT Q600 Instrument. Thermogravimetric curves performed under a synthetic air atmosphere. Approximately 200 mg (350 mg, including sample holder) of samples were loaded to a platinum.
